# Characterizing circulating rare cells in peripheral blood for detecting and monitoring multiple myeloma and precursor states

**DOI:** 10.1038/s41698-025-01175-2

**Published:** 2025-12-02

**Authors:** Stephanie N. Shishido, Jeremy Mason, Mohamed Kamal, Sonia Maryam Setayesh, Amishi U. Vora, David Berrios, Luz Yurany Moreno Rueda, Krina Patel, Elisabet E. Manasanch, Robert Z. Orlowski, Peter Kuhn

**Affiliations:** 1 Convergent Science Institute for Cancer, Michelson Center, University of Southern California, Los Angeles, CA USA; 2 Institute of Urology, Catherine & Joseph Aresty Department of Urology, Keck School of Medicine, University of Southern California, Los Angeles, CA USA; 3 Norris Comprehensive Cancer Center, Keck School of Medicine, University of Southern California, Los Angeles, CA USA; 4 Department of Lymphoma and Myeloma, Division of Cancer Medicine, University of Texas MD Anderson Cancer Center, Houston, TX USA; 5 Department of Experimental Therapeutics, Division of Cancer Medicine, University of Texas MD Anderson Cancer Center, Houston, TX USA; 6 Department of Biomedical Engineering, Viterbi School of Engineering, University of Southern California, Los Angeles, CA USA; 7 Department of Aerospace and Mechanical Engineering, Viterbi School of Engineering, University of Southern California, Los Angeles, CA USA; 8 Department of Biological Sciences, Dornsife College of Letters, Arts, and Sciences, University of South-ern California, Los Angeles, CA USA

**Keywords:** Tumour heterogeneity, Myeloma

## Abstract

Multiple myeloma (MM) arises from abnormal plasma cells (PCs) progressing from precursor states, including monoclonal gammopathy of undetermined significance (MGUS) and smoldering multiple myeloma (SMM). Understanding this transition and progression to overt MM requires improved non-invasive strategies. We employed a liquid biopsy approach to detect and characterize circulating PCs across disease states in 68 patients (MGUS = 11, SMM = 21, NDMM = 19, RRMM = 17) using multi-channel immunofluorescence staining and machine learning-assisted rare event detection. PCs were identified by CD138 and B-cell maturation antigen (BCMA) expressions, with distinct phenotypic subpopulations stratifying disease states. The D | CD138 | BCMA-Memb phenotype was the most predictive, with incidence increasing from MGUS to SMM and overt MM (p < 0.005). Multivariate modeling distinguished precursors from overt disease with 86% accuracy. Shifts in BCMA and CD45 expression suggested immune cell profile alterations with progression and treatment. These findings underscore PB-based liquid biopsy as a promising tool for MM detection and monitoring, revealing circulating PC heterogeneity.

## Introduction

Multiple myeloma (MM) is a hematologic malignancy caused by the clonal proliferation of plasma cells (PCs). In 2024 alone, MM was projected to account for approximately 35,780 new cases and 12,540 deaths in the United States^1^. The clinical trajectory of MM involves two precursor states: monoclonal gammopathy of undetermined significance (MGUS) and smoldering multiple myeloma (SMM). Over time, these precursor states can progress to overt symptomatic MM (newly diagnosed; NDMM) or relapsed/refractory MM (RRMM). Diagnosing progression to MM early remains a significant challenge due to its mostly asymptomatic nature in initial stages and the disease’s inherent heterogeneity^2–4^. Moreover, despite recent advancements, such as B-cell maturation antigen (BCMA)-targeted therapies, MM continues to exhibit high relapse rates^5–8^. Improved methods are needed to monitor disease progression and treatment efficacy.

Traditionally, MM diagnosis and monitoring rely on bone marrow (BM) evaluation via aspirate and biopsy^9^. This procedure is invasive with the potential for complications thus being impractical for repetitive disease evaluation. Additionally, it is usually a blind sample from a random site at the iliac crest which does not capture the heterogeneity of the BM due to the patchy myeloma infiltration. In contrast, peripheral blood (PB) liquid biopsy offers a promising, minimally invasive alternative for disease detection and monitoring by analyzing circulating tumor-related analytes released into the blood stream from the BM. For example, cell-free DNA (cfDNA) analysis has shown potential in monitoring minimal residual disease (MRD) and predicting progression-free survival in advanced MM^10–12^. Similarly, the detection of circulating myeloma cells and exosomes in PB have been correlated with disease progression and poor overall survival^13–19^ even though circulating myeloma cells are found at a lower incidence in the PB than the BM^20,21^. PB has the potential to provide dynamic insights into disease burden from the entirety of the BM compartment, progression, and molecular characteristics, enabling real-time surveillance and facilitating personalized treatment strategies.

Traditional methods for detecting circulating myeloma cells, such as flow cytometry and enrichment-based techniques, are limited in sensitivity and may overlook clinically significant subpopulations of circulating myeloma cells, such as CD138-low expressing cells with greater clonogenic potential. To address these limitations, we utilized the High-Definition Single Cell Assay (HDSCA) workflow as a non-enrichment liquid biopsy tool to analyze PB samples from 68 patients across the MM disease spectrum, leveraging an immunofluorescence assay targeting CD138, BCMA, and CD45 to identify a diverse population of tumor-associated circulating PCs and differentiate disease states. The findings highlight the potential of PB analyses to enhance MM detection and monitoring, particularly in precursor stages, such as MGUS and SMM, thus providing a comprehensive, minimally invasive method for disease surveillance.

## Results

### Patient demographics and clinical baseline

A total of 68 blood draws were collected for this study with 1 sample per patient. All patients enrolled underwent diagnostic evaluation of their disease state: 11 MGUS, 21 SMM, 19 NDMM, and 17 RRMM patients. Patient demographics and clinical data are provided in Table 1 (additional details from clinical workup are provided in Supplementary Table 1).

**Table 1:** Clinical and demographic data for disease cohort

Variable, Category	ALL	MGUS (*n* = 11,16%)	SMM (*n* = 20, 29%)	NDMM (*n* = 19, 28%)	RRMM (*n* = 18, 26%)
**Age, years**	*n* = 68	*n* = 11	*n* = 20	*n* = 19	*n* = 18
**Median (min-max)**	64 (38–88)	70 (38–78)	61 (42–74)	63 (49–85)	65 (43–88)
**Gender,** ***n*** **(%)**					
**Male**	32 (47.06)	3 (27.27)	7 (35)	10 (52.63)	12 (66.67)
**Female**	36 (52.94)	8 (72.73)	13 (65)	9 (47.37)	6 (33.33)
**Race, n (%)**					
**American Indian or Alaskan Native**	1 (1.47)	0 (0)	0 (0)	0 (0)	1 (5.56)
**Asian**	1 (1.47)	0 (0)	0 (0)	0 (0)	1 (5.56)
**Black or African American**	8 (11.76)	0 (0)	2(10)	4 (21.05)	2 (11.11)
**Native Hawaiian or Other Pacific Islander**	1 (1.47)	0 (0)	1 (5)	0 (0)	0 (0)
**Other**	3 (4.41)	0 (0)	1 (5)	0 (0)	2 (11.11)
**White or Caucasian**	54 (79.41)	11 (100)	16 (80)	15 (78.95)	12 (66.67)
**Ethnicity, n (%)**					
**Hispanic or Latino**	10 (14.71)	0 (0)	0 (0)	5 (26.32)	5 (27.78)
**Not Hispanic or Latino**	57 (83.82)	11 (100)	20 (100)	13 (68.42)	13 (72.22)
**Patient Refused**	1 (1.47)	0 (0)	0 (0)	1 (5.26)	0 (0)
**ECOG Score, n (%)**					
**0**	6 (8.82)	1 (9.09)	4(20)	0 (0)	1 (5.56)
**1**	48 (70.59)	10 (90.91)	15 (75)	14 (73.68)	9 (50.00)
**Not Available**	14 (20.59)	0 (0)	1 (5)	5 (26.32)	8 (44.44)
**M spike Value (SPEP), gm/dL**					
**Median (min-max)**	0.95 (0-8.9)	0.4 (0-1.6)	1.3 (0-3.9)	1.9 (0-8.9)	0.45 (0-7.5)
**FLOW Aberrant PCs from total analyzed, %**					
**Median (min-max)**	95.1 (0-100)	40 (0-97)	94.5 (42.9-99.7)	99 (11-100)	94.5 (0-100)
**Alive, n (%)**					
**Yes**	60 (88.24)	11 (100)	20 (100)	17 (89.47)	12 (66.67)
**No**	8 (11.76)	0 (0)	0 (0)	2 (10.53)	6 (33.33)

N/A: not reported or available

### Identification and enumeration of cells of interest

**Fig. 1:**
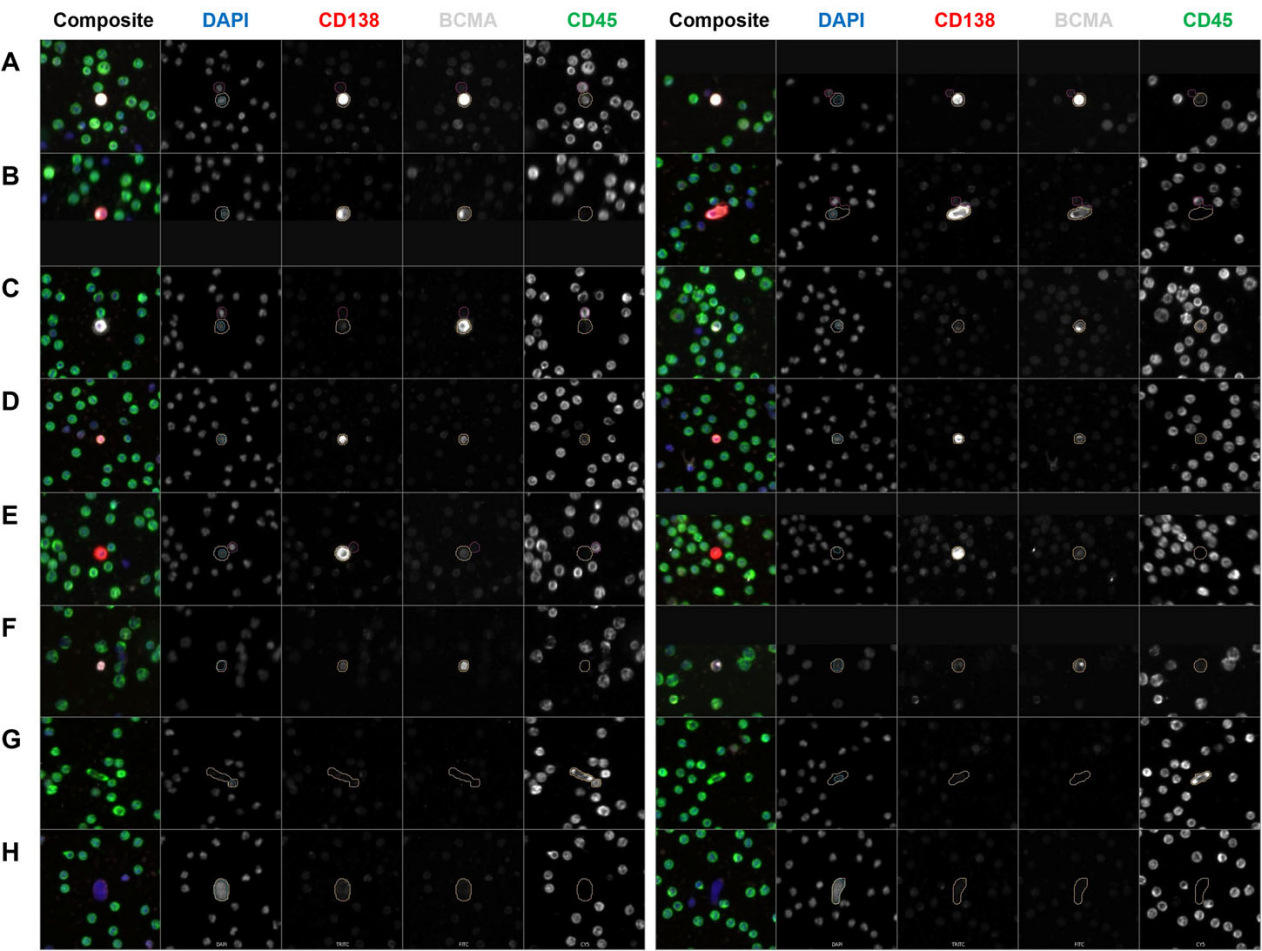
Representative gallery of circulating rare events detected in the liquid biopsy of patients diagnosed with precursor or overt disease. The composite image and each individual biomarker channels are provided. The cell groups were classified into 12 classes based on the combination of DAPI (D) and 3 markers: CD138, BCMA (membrane [Memb] and perinuclear [Peri] localization), and CD45. **A** D | CD138 | BCMA | CD45, **B** D | CD138 | BCMA, **C** D | BCMA | CD45, **D** D | CD138 | CD45, **E** D | CD138, **F** D | BCMA, **G** D | CD45, **H** DAPI-only. BCMA membrane and perinuclear localization examples provided in the left and right columns, respectively. Images taken at 100x magnification. Blue: DAPI, Red: CD138, White: BCMA, Green: CD45.

Circulating rare cells of interest were identified and classified based on their biomarker signal expression and localization following our four-channel immunofluorescence staining corresponding to D, CD138, BCMA, and CD45 (Fig. 1). Circulating PCs were defined by the expression of CD138 and/or BCMA.

**Fig. 2:**
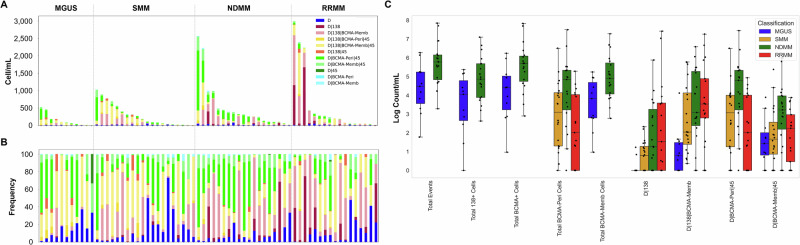
Circulating rare cells detected in the PB of patients diagnosed with MGUS, SMM, NDMM, or RRMM. **A** Enumeration and **B** frequency of circulating rare cells based on channel classification. **C** Boxplot of cellular distribution per disease state for comparisons that are significantly different (p-value < 0.05). All cellular comparisons are provided in Supplementary Fig. 1. Black line is median, boxed area is the interquartile range (IQR), error-bars show the complete range of the data. D: DAPI, 138: CD138, 45: CD45, Peri: perinuclear localization of BCMA, Memb: membrane bound BCMA.

PB samples taken after diagnosis were compared across the disease spectrum to determine the presence of circulating PCs (Fig. 2). Table 2 shows the cell types with significant differences between disease states. MGUS was distinguished from NDMM by total cells (p-value = 0.0150), total CD138^+^ (p-value = 0.0409), total BCMA^+^ (p-value = 0.0190), total BCMA-Memb (p-value = 0.0169), D | CD138 (p-value = 0.0081), D | CD138 | BCMA-Memb (p-value = 0.0021), and D | BCMA-Memb|CD45 (p-value = 0.0169) cells. SMM was distinguished from NDMM by total BCMA-Peri (p-value = 0.0294), D | BCMA-Peri|CD45 (p-value = 0.0190) and D | BCMA-Memb|CD45 (p-value = 0.0229) cells.

**Table 2:** Statistically significant comparisons of PB rare cells between disease states

Groups (1 vs. 2)	Event Type	P-value	Mean 1	Mean 2
MGUS vs. NDMM	D | 138 | BCMA-Memb	0.0021	1.0026	3.4532
	D | 138	0.0081	0.1888	1.8354
	Total Cells	0.0150	4.3766	5.7071
	D | BCMA-Memb|45	0.0169	1.5441	3.0236
	Total BCMA-Memb Cells	0.0169	3.5482	4.9519
	Total BCMA^+^ Cells	0.0190	4.1197	5.5063
	Total 138^+^ Cells	0.0409	3.4876	4.8627
MGUS vs. SMM	D | 138 | BCMA-Memb	0.0044	1.0026	2.7346
NDMM vs. RRMM	D | BCMA-Peri|45	0.0036	0.9558	0.4274
	Total BCMA-Peri Cells	0.0043	4.1966	2.2193
	D | BCMA-Memb|45	0.0324	3.0236	1.8540
MGUS vs. RRMM	D | 138 | BCMA-Memb	0.0038	1.0026	3.6084
	D | 138	0.0084	0.1888	2.3244
SMM vs. NDMM	D | BCMA-Peri|45	0.0190	2.7677	4.1726
	D | BCMA-Memb|45	0.0229	1.8338	3.0236
	Total BCMA-Peri Cells	0.0294	2.9458	4.9519

*D* DAPI, 138: CD138, 45: CD45, *Peri* perinuclear localization of BCMA, *Memb* membrane bound BCMA. Two-sided Wilcoxon rank sum test with significance determined at a P-value < 0.05.

There was one specific cellular phenotype that was the most significant at stratifying the disease states in which incidence increased with disease status: D | CD138 | BCMA-Memb. These cells were able to stratify MGUS from the overt disease states (NDMM [p-value = 0.0021] and RRMM [p-value = 0.0038]), while also presenting at a significantly higher count in SMM than MGUS (p-value = 0.0044). This indicates the potential to monitor transition between precursor states and overt disease using PB. Interestingly, NDMM and RRMM were stratified by total BCMA-Peri (p-value = 0.0043), D | BCMA-Memb|CD45 (p-value = 0.0324), and D | BCMA-Peri|CD45 cells (p-value = 0.0036), all of which NDMM samples had a significantly higher incidence of cells compared to RRMM. This suggests a potential shift in the cellular profile after treatment. Overall, this data indicates there are unique PCs present in the PB across the disease states.

### Cellular morphometrics

**Fig. 3:**
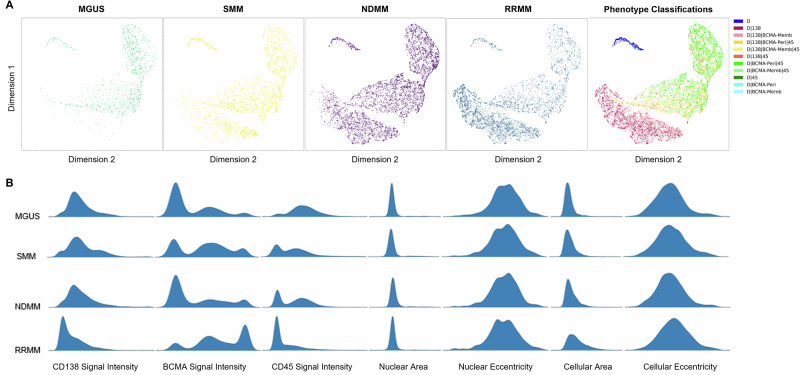
Graphical representation of the different channel-type rare cells/mL between disease states. **A** Morphology UMAP plot of rare cells by channel-type classification and separated by disease state: MGUS, SMM, NDMM, RRMM. **B** Probability density distribution plots for select morphometric parameters across channel-type classifications of median CD138 signal intensity, median BCMA signal intensity, median CD45 signal intensity, nuclear area, nuclear eccentricity, cellular area, and cellular eccentricity.

The heterogeneity of the rare cell populations detected in PB across different disease states was analyzed using cellular morphometrics. Two-dimensional UMAP plots are presented in Fig. 3A, where each rare cell is represented as a single point. These plots illustrate the progression of CD138^+^ PCs, which increase in abundance as the disease advances. Additional distributions of cellular and nuclear area, eccentricity, and median channel intensities for each disease state are shown in Fig. 3B, further highlighting the morphological diversity and variability of the rare cell populations across disease progression. For example, the observed total BCMA signal intensity, which includes both membrane and perinuclear contributions, reveals three distinct peaks. While the density plot does not distinguish signal localization, imaging suggests that these peaks may correspond to functionally distinct BCMA^+^ PC subtypes, with a notable transition to higher signal intensity in RRMM. Conversely, observed CD45 signal intensity shifts toward lower expression levels as the disease status becomes more aggressive. Interestingly, the nuclear and cellular areas and eccentricities remain relatively stable, with only minor observable variations from precursor to overt disease states. These findings underscore the morphological and functional complexity of the circulating PC populations as MM progresses.

### Correlations between the circulating cell subsets and clinical metrics

Correlation analyses were performed to investigate potential clinical associations between circulating rare cells identified in PB samples and the various clinical data metrics, which included the diagnostic parameters (based on BM) and survival information. The significant correlations observed are summarized in Table 3. No significant associations were found for variables, such as gender, ethnicity, or race.

**Table 3:** Significant correlations between the clinical/demographic variables and the rare cells identified by HDSCA in the PB samples collected from patients with precursor states and overt MM

HDSCA analyte	Clinical variable	Method	Correlation value	P-value
D | 138 | BCMA-Memb	Monosomy 13/ loss RB1/ Del 13	Wilcoxon	−4.1405	0.0000
D | 138 | BCMA-Memb	PC percentage in left core	Spearman	0.5085	0.0001
D | 138 | BCMA-Memb	FLOW aberrant PCs from total analyzed percent	Spearman	0.4317	0.0003
D | 138	serum free light chain ratio	Spearman	0.4173	0.0004
D | 138 | BCMA-Memb	PC percentage in left aspirate	Spearman	0.4602	0.0007
D | 138	Bence Jones	Spearman	0.4398	0.0009
D | 138 | BCMA-Memb	Bence Jones	Spearman	0.4237	0.0014
D | 138 | BCMA-Memb	serum free light chain ratio	Spearman	0.3636	0.0025
D | 138 | BCMA-Memb	PC percentage in right core	Spearman	0.3996	0.0025
D | 138 | BCMA-Memb	PC percentage in right aspirate	Spearman	0.3729	0.0046
Total 138^+^ Cells	Del of CDKN2C (1q32)	Wilcoxon	−2.7221	0.0065
Total BCMA-Memb Cells	Del of CDKN2C (1q32)	Wilcoxon	−2.6075	0.0091
Total BCMA^+^ Cells	Age	Spearman	−0.3124	0.0101
D | 138	CD27 simplified	Wilcoxon	2.5666	0.0103
Total 138^+^ Cells	Monosomy 13/ loss RB1/ Del 13	Wilcoxon	−2.5580	0.0105
D | 138	Monosomy 13/ loss RB1/ Del 13	Wilcoxon	−2.5218	0.0117
Total Events	Del of CDKN2C (1q32)	Wilcoxon	−2.4928	0.0127
D | 138 | BCMA-Memb	Gain CKS1B (1q21)-trisomy 1	Wilcoxon	−2.4855	0.0129
D | 138 | BCMA-Memb	IgM	Spearman	−0.2966	0.0148
Total BCMA-Memb Cells	Monosomy 13/ loss RB1/ Del 13	Wilcoxon	−2.4279	0.0152
D | 138	Alive	Wilcoxon	2.4267	0.0152
D | 138 | BCMA-Memb	IgA	Spearman	−0.2914	0.0167
D | BCMA-Peri|45	Gain FGFR3 /trisomy 4	Wilcoxon	−2.3905	0.0168
Total BCMA-Peri Cells	Gain FGFR3 /trisomy 4	Wilcoxon	−2.3905	0.0168
Total Events	Age	Spearman	−0.2863	0.0188
Total BCMA^+^ Cells	Del of CDKN2C (1q32)	Wilcoxon	−2.3209	0.0203
D | BCMA-Peri	Gain FGFR3 /trisomy 4	Wilcoxon	−2.3108	0.0208
D | BCMA-Memb|45	Gain FGFR3 /trisomy 4	Wilcoxon	−2.3108	0.0208
D | 138	IgM	Spearman	−0.2818	0.0209
D | 138	urine immunofixation light chain	Wilcoxon	2.2402	0.0251
D | BCMA-Memb	Gain 17p/TP53 - trisomy 17	Wilcoxon	−2.2381	0.0252
D | 138 | BCMA-Peri|45	Gain FGFR3 /trisomy 4	Wilcoxon	−2.2311	0.0257
Total BCMA-Memb Cells	Bence Jones	Spearman	0.3021	0.0264
D | 138	PC percentage in left core	Spearman	0.3094	0.0271
Total Events	Bence Jones	Spearman	0.2980	0.0286
Total BCMA^+^ Cells	Bence Jones	Spearman	0.2971	0.0291
D | 138 | BCMA-Memb|45	Alive	Wilcoxon	−2.1753	0.0296
Total 138^+^ Cells	Bence Jones	Spearman	0.2960	0.0298
Total BCMA-Memb Cells	Age	Spearman	−0.2654	0.0300
D | 138 | BCMA-Memb|45	Age	Spearman	−0.2619	0.0322
Total BCMA-Peri Cells	Age	Spearman	−0.2607	0.0331
D | 138	CD45	Wilcoxon	−2.1301	0.0332
D | 138 | 45	Alive	Wilcoxon	−2.1270	0.0334
D | 138	CD19 simplified	Wilcoxon	2.1190	0.0341
D | 138	IgA	Spearman	−0.2592	0.0342
Total Events	Gain FGFR3 /trisomy 4	Wilcoxon	−2.1116	0.0347
Total BCMA^+^ Cells	Gain FGFR3 /trisomy 4	Wilcoxon	−2.1116	0.0347
D | BCMA-Memb	Gain FGFR3 /trisomy 4	Wilcoxon	−2.0917	0.0365
D | 138 | BCMA-Memb	gain CCND1/MYEOV/IGH -trisomy 11	Wilcoxon	−2.0781	0.0377
D | 138 | BCMA-Peri|45	serum free light chain ratio	Spearman	−0.2538	0.0382
D	serum free light chain ratio	Spearman	−0.2527	0.0391
D | 138 | BCMA-Memb	Del of CDKN2C (1q32)	Wilcoxon	−2.0487	0.0405
Total BCMA-Peri Cells	serum immunofixation	Wilcoxon	2.0421	0.0411
D | 138 | 45	gain CCND1/MYEOV/IGH -trisomy 11	Wilcoxon	−2.0393	0.0414
D | 138	cyKAPPA	Wilcoxon	2.0305	0.0423
D | BCMA-Memb|45	Del of CDKN2C (1q32)	Wilcoxon	−1.9914	0.0464
Total 138^+^ Cells	PC percentage in left core	Spearman	0.2800	0.0466
Total 138^+^ Cells	Gain CKS1B (1q21)-trisomy 1	Wilcoxon	−1.9787	0.0478
D | BCMA-Peri|45	serum immunofixation	Wilcoxon	1.9762	0.0481
D | BCMA-Peri|45	Age	Spearman	−0.2416	0.0489

*D* DAPI, 138: CD138, 45: CD45, *Peri* perinuclear localization of BCMA, *Memb* membrane bound BCMA, *FLOW* flow cytometry. Two-sided Wilcoxon rank sum test or Spearman correlation analyses with significance determined at a P-value < 0.05.

The D | CD138 | BCMA-Memb phenotypic cells were the most commonly correlated PB analyte with most of the BM diagnostic measurements, which lead to the patient diagnosis. Additionally, the D | CD138, D | CD138 | BCMA-memb|CD45, D | CD138 | CD45 phenotypes correlated with disease survival. Interestingly, the total cells and total BCMA^+^ cells correlated with age, which suggests BCMA phenotypic changes in the immune cell profile independent of disease state as the body ages.

### Patient level prediction

To determine if the PB could be used to detect precursor states from overt disease in an individual, we conducted both univariate and multivariate analyses (Supplementary Table 2) which indicated that the D | CD138 PCs were the highest single predictor (accuracy = 79%) and most important cellular phenotype for both decision tree and random forest (accuracy = 86%) in determining progression. When considering stratification of precursor states with only NDMM, the D | CD138 PCs was the highest predictor (accuracy = 80%). For the multivariate model, the decision tree and random forest predicted with 70% accuracy, both indicating the D | BCMA-Memb|CD45 cells as the most important feature. Similarly, we wanted to determine if the distinct precursor states could be predicted by PB analytes. The D | CD138 | BCMA-Memb PCs had a 71% accuracy in predicting the transition from MGUS to SMM for the univariate model and was the most important predictor in the decision tree model (accuracy = 71%). Together these findings highlight the potential utility of PB PCs as predictors of disease with tailored modeling approaches for optimized accuracy based on the clinical question, underscoring the value of heterogenous PCs in early detection and monitoring of disease status.

## Discussion

In this proof-of-concept study, we have demonstrated the feasibility of detecting circulating rare cells, including a variety of PCs, in the blood of patients with MM and its precursor conditions. Slide-based immunofluorescence methods similar to what we presented here have detected circulating myeloma PCs in 19, 25, and 80% of MGUS, SMM, and NDMM, respectively^22–25^. Using a non-enrichment approach, we have characterized the spectrum of PCs (malignant and non-malignant) and rare cells to show the transition of cells in the PB across disease states with a prediction accuracy of 86% by multivariate analysis. Further, the correlations between PC subtypes detected in the PB and BM flow cytometry measurements support a biologically relevant link between compartments. These exploratory results underscore the transformative potential of the PB liquid biopsy as a non-invasive diagnostic and monitoring tool. We identified cell types that not only stratify disease stages but also correlate with clinical parameters by leveraging multi-channel immunofluorescence and rigorous phenotypic characterization. Unlike traditional diagnostic methods that rely on BM, which are invasive, painful, and associated with potential complications, the PB offers a quick, minimally invasive alternative. By minimizing discomfort and the risks associated with invasive sampling, PB can enhance patient compliance and facilitate more frequent monitoring, which is critical for managing a disease with a high risk of progression and relapse.

Our findings highlight the potential utility of specific circulating cell subsets, particularly D | CD138 | BCMA-Memb phenotypic PCs, in differentiating between precursor states (MGUS and SMM) and overt disease (NDMM and RRMM). This cellular phenotype demonstrated the most significant ability to stratify disease states, with incidence progressively increasing from MGUS to SMM and overt disease. Notably, D | CD138 | BCMA-Memb PCs also distinguished MGUS from SMM, emphasizing their potential utility in identifying subtle transitions within precursor states (p-value = 0.0044). Importantly, the ability to identify myeloma PCs in precursor conditions like MGUS and SMM presents an unprecedented opportunity for early detection and intervention. A prior study found that patients with MGUS could be divided into two groups, one likely to progress to advanced disease and another that remains stable over time^26^. Univariate and multivariate prediction analyses achieved moderate accuracy (71%), reflecting the phenotypic overlap and biological uncertainty in this early-stage transition, which mirrors the clinical overlap and the challenge of defining progression within precursor states, which underscores a critical area for future research. The transition from these precursor states to symptomatic MM is typically recognized only after significant organ damage or clinical symptoms emerge^27,28^, but a PB-based approach could shift this paradigm by enabling real-time tracking of disease evolution. Early identification of patients at high risk for progression could allow clinicians to implement preventative or therapeutic measures earlier, potentially delaying or preventing symptomatic MM, ultimately improving patient outcomes by reducing disease burden and preserving organ function. This is especially important in SMM where registrational trials are underway that could lead to an FDA approved treatment that will revolutionize our standard of care approach to precursor states.

It is important to note that other phenotypic PC classifications provided insights into the biological transition. BCMA signal intensity revealed three distinct subtypes, with higher expression in RRMM, while CD45 intensity decreased as disease status became more aggressive, highlighting the functional complexity of circulating PCs in MM. These findings suggest that treatment impacts the cellular profile, with RRMM samples showing a distinct shift compared to treatment-naïve NDMM samples. Such shifts in cellular profiles post-treatment could provide insights into mechanisms of treatment resistance and guide therapeutic decision-making. Correlation analyses further revealed that total cells and BCMA^+^ PCs were associated with age, indicating BCMA-related phenotypic changes in immune cell profiles that may occur independently of disease state. These findings suggest that age-related immune alterations could contribute to MM progression and warrant further investigation.

Moreover, PB facilitates longitudinal monitoring, providing a dynamic view of disease progression, therapeutic response, and the emergence of resistance. By allowing frequent sampling and analysis of PCs, clinicians can gain insights into the molecular and cellular changes driving the disease, enabling adaptive treatment strategies tailored to individual patient needs. This capability is particularly valuable in MM as a highly heterogeneous disease composed of various molecular subgroups, each characterized by an assortment of genomic alterations with evolving treatment landscapes^29,30^, including therapies targeting BCMA. Detailed characterization of the genetic, epigenetic, and phenotypic profiles of these myeloma PCs could shed light on the pathways driving disease initiation, progression, and resistance to therapy. Such insights could guide the identification of novel therapeutic targets and inform the development of more effective treatment strategies tailored to specific disease subtypes or progression patterns. Previous studies assessing circulating MM cells typically use flow cytometry and have shown limitations in the utility of the PB compared to BM, especially in detecting MRD^31–33^. The method used here provides an unbiased cellular detection to profile rare cells with the potential for downstream molecular analyses^34–36^, showing potential clinical promise for PB as a tool to assess treatment efficacy and detect early signs of relapse, thus ensuring timely intervention and optimizing therapeutic outcomes.

While a direct comparison to established platforms, such as CellSearch or flow cytometry would provide valuable context, it was not feasible within the scope of this study due to limitations in sample volume, IRB protocol constraints, and distinct pre-analytical requirements for each platform. Nonetheless, our approach offers several advantages that support its complementary and, in some contexts, superior analytical value. Most notably, our method demonstrates increased sensitivity in detecting rare PC subsets, including phenotypically diverse or low-abundance populations that may be missed by conventional marker-restricted approaches. Unlike flow cytometry, which typically requires immediate processing and may compromise cell integrity for downstream analyses, our platform preserves rare cells in a state suitable for single-cell genomic and proteomic profiling, allowing for a broader analytical scope. The platform used here is also antigen-agnostic and adaptable to diverse surface markers, enabling the detection of heterogeneous or low-abundance cell populations that may escape detection by marker-restricted platforms, such as CellSearch. Additionally, the workflow accommodates up to 48 h from blood draw to processing, offering logistical flexibility for multi-center or clinically integrated studies. We are currently planning future studies to assess the degree of overlap and complementarity between technologies, which will further define the unique contributions and potential clinical utility of our assay in the context of PC and rare cell analysis.

Despite its promise, this study has notable limitations that warrant further investigation. The relatively small sample size per disease state limits the generalizability of the findings, emphasizing the need for validation in larger and more diverse patient cohorts. Additionally, while the detection and characterization of myeloma PCs in blood samples represent significant advancements, further refinement of the techniques is essential to enhance assay sensitivity and specificity. Improving these methodologies will be critical to capturing the full spectrum of disease heterogeneity and ensuring robust clinical applicability. By enabling non-invasive, longitudinal tracking of disease dynamics, liquid biopsies may offer a patient-centered approach that enhances care delivery. Future analyses at the cohort level will explore this through serial sampling across patients. Furthermore, the molecular insights gained from these analyses can deepen our understanding of MM biology, paving the way for innovative therapeutic interventions and improved outcomes for patients. These findings underscore the importance of continued research to optimize liquid biopsy techniques and validate their utility across diverse clinical settings.

The main limitation of this study is the moderate follow-up time, which restricts our ability to correlate findings with standard survival metrics, which is a key objective of ongoing and future research. The follow-up time for patients was highly variable, reflecting the real-world nature of the cohort. Specifically, follow-up durations ranged from 0 days (in newly diagnosed patients) to 6 years, depending on the timing of enrollment and disease stage at the time of sample collection. Survival analyses, such as Kaplan-Meier curves or other modeling approaches, will be essential to better assess the prognostic significance of circulating PC phenotypes. Given that survival remains the gold standard for biomarker validation, separating survival analyses from other clinical metrics will be crucial to fully understand the impact of circulating PCs on patient outcomes, paving the way for their use in clinical decision-making. Furthermore, while our study focused on circulating PCs, integrating other liquid biopsy analytes, such as cell-free DNA or extracellular vesicles, could provide a more comprehensive picture of disease evolution. Improved models incorporating additional biomarkers or longitudinal data may enhance diagnostic accuracy, stratify patients based on risk, and identify therapeutic targets, further advancing the personalized care of MM. While our findings are promising, further work is needed to establish sensitivity, specificity, and clinical thresholds in larger, independent cohorts.

## Methods

### Study design

The study recruited and enrolled patients following an approved protocol (PA18-1073) from the Institutional Review Board (IRB) at The University of Texas MD Anderson Cancer Center (MDACC). All study participants have provided written informed consent in accordance with the principles of the Declaration of Helsinki. Patients underwent standard diagnostic tests, including a BM aspirate and core needle biopsy, bloodwork, and whole-body imaging. The study collected matched PB and BM specimens from 68 patients (11 MGUS, 21 SMM, 19 NDMM, 17 RRMM). Paired BM samples were taken for clinical workup only. RRMM patients have undergone one or more lines of therapy.

### Sample acquisition and processing

All blood samples (8 mL) were collected using anti-coagulated preservative tubes (Cell-Free DNA blood collection tube, Streck, La Vista, NE, USA) at MDACC and shipped to the USC Michelson Convergent Science Institute in Cancer (CSI-Cancer) via FedEx overnight at controlled room temperature using validated, temperature-stabilizing containers, and processed within 48 h post-draw, as previously described^35,37–39^. Upon sample arrival, a complete blood cell count was taken and red blood cells lysed. All remaining nucleated cells were plated as a monolayer at approximately 3 million cells per custom cell adhesive glass slide (Marienfeld, Lauda, Germany). Slides were subsequently incubated in 7% BSA, dried, and stored at −80 °C.

### Immunofluorescent staining

Two slides per sample were thawed prior to immunofluorescent (IF) staining using an IntelliPATH FLX™ autostainer (Biocare Medical LLC, Irvine, CA, USA) as previously described^35^. All steps were conducted at room temperature. Slides were fixed with 2% paraformaldehyde for 20 min, blocked with 10% goat serum for 30 min, and incubated with a primary antibody mix consisting of CD138 (2 µg/mL, Exbio, clone A-38, cat# 10-520-C100, Vestec, Czech Republic), CD45 Alexa647 (1.6 µg/mL, AbD Serotec, cat# MCA87A647, Raleigh, NC, USA), and BCMA (2.5 µg/mL, Abcam, cat# ab253242, Cambridge UK) for 1 h. Next, slides were blocked again with 10% goat serum for 1 h prior to incubation with secondary antibodies AlexaFluor555 goat anti-mouse (1:500, Invitrogen, cat# A21127, Waltham, MA, USA), AlexaFluorPLUS488 goat anti-rabbit (1:500, Invitrogen, cat# A32731, Waltham, MA, USA), and DAPI (4’,6-diamidino-2-phenylindole; D) for 40 min. Slides were mounted with a glycerol-based media, coverslipped, and sealed for downstream imaging and technical analysis.

### Image acquisition, processing, and cell enumeration

Automated high-throughput fluorescence scanning microscopy at 100X magnification was employed for the imaging of 2304 frames across each slide as previously described^35,37^. The gain and exposure times for all channels were normalized by the scanner software.

Rare event detection was conducted through a customized algorithm Outlier Clustering Unsupervised Learning Automated Report (OCULAR), which was previously used in analysis of liquid biopsy samples for detection of rare events in carcinomas^40–42^. This method was modified for the purposes of this study in MM disease progression. In short, the approach utilizes principal component analysis (PCA) and hierarchical clustering to identify small clusters of cells or individual cells that significantly deviate from the median cell based on computational distance. To further refine this process and eliminate technical artifacts, we developed a machine learning classification model to determine the biological relevance of cells. A histogram gradient boosting algorithm was implemented with k-fold cross-validation across 1000 iterations to identify the top-performing models. Additionally, hyperparameter grid searches were conducted on morphometric features to pinpoint subsets that optimized model accuracy. Prediction confidence for each cell was used as a threshold to balance sensitivity (accurately identifying biologically relevant events) and specificity (accurately excluding irrelevant events). The model was developed from manually curated data of 19 samples (24,300 individual cells).

Slides with manual cell-level annotations were divided into training and testing sets, while non-annotated slides were utilized in downstream model development to test performance. The cell groups were classified into 12 classes based on the combination of three markers: CD138, BCMA (membrane [Memb] and perinuclear [Peri] localization), and CD45. The channel classification models were developed from manual cellular classifications from 11 samples (2906 individual cells). Cells are characterized by their IF expression in each of the channels and referred to by only their positive markers. The supervised machine learning algorithms were trained to distinguish between groups using the full feature space, allowing it to capture complex, non-linear patterns that may not be evident through traditional threshold-based or univariate statistical analyses.

The OCULAR rare cells detected were automatically curated with the previously mentioned machine learning model set at 90% prediction confidence to reduce noise and subsequently characterized by their IF channel expression. Using the cell count taken at processing and the cell count per slide analyzed, the final cell enumerations were able to be reported as cells per mL.

### Statistical analysis

Non-parametric statistical two-sided tests were used to determine correlations between detected rare events in the liquid biopsy samples and clinical parameters. We utilized the Wilcoxon rank sum test (categorical) and Spearman’s rank correlation (continuous and ordinal) to analyze correlations between rare event enumerations and clinical data elements. P-values below 0.05 were considered statistically significant. Each comparison was analyzed independently. Cellular morphometrics were used to investigate the heterogeneity of the rare-event population. We visualized cell heterogeneity via two-dimensional UMAPs (Uniform Manifold Approximation and Projection) and morphometric probability distribution plots.

## Supplementary information

Supplementary information

## Data Availability

All data discussed in this manuscript are included in the main manuscript text or supplementary materials. The imaging data are available through the BloodPAC Data Commons, Accession ID “BPDC000152” (https://data.bloodpac.org/discovery/BPDC000152).

## Code availability

All code developed is standard and easily reproducible.
