# Supplementary material for: Characterizing circulating rare cells in peripheral blood for detecting and monitoring multiple myeloma and precursor states
Source: NPJ Precis Oncol. 2025 Dec 2;9:388. doi: 10.1038/s41698-025-01175-2 (PMC12672686; doi:10.1038/s41698-025-01175-2)
Supplement: Supplementary file 1 — Supplementary information [file 41698_2025_1175_MOESM1_ESM.pdf]

Supplemental Information

Supplemental Table 1. Clinical and demographic data for disease cohort. N/A: not reported or available, FLOW: flow cytometry.

Variable, Category	Value	Variable, Category	Value
**Age, years**		**CD38, n (%)**	
**Median (min-max)**	64 (38-88)	**Positive**	60 (88.24)
**Gender, n (%)**		**Negative**	4 (5.88)
**Male**	32 (47.06)	**N/A**	4 (5.88)
**Female**	36 (52.94)	**CD45, n (%)**	
**Race, n (%)**		**Positive**	28 (41.18)
**American Indian or Alaskan Native**	1 (1.47)	**Negative**	36 (52.94)
**Asian**	1 (1.47)	**N/A**	4 (5.88)
**Black or African American**	8 (11.76)	**CD56, n (%)**	
**Native Hawaiian or Other Pacific Islander**	1 (1.47)	**Positive**	49 (72.06)
**Other**	3 (4.41)	**Negative**	15 (22.06)
**White or Caucasian**	54 (79.41)	**N/A**	4 (5.88)
**Ethnicity, n (%)**		**CD81, n (%)**	
**Hispanic or Latino**	10 (14.71)	**Positive**	13 (19.12)
**Not Hispanic or Latino**	57 (83.82)	**Negative**	50 (73.53)
**Patient Refused**	1 (1.47)	**N/A**	5 (7.35)
**ECOG Score, n (%)**		**CD117, n (%)**	
**0**	6 (8.82)	**Positive**	34 (50)
**1**	48 (70.59)	**Negative**	30 (44.12)
**Not Available**	14 (20.59)	**N/A**	4 (5.88)
**Diagnosis, n (%)**		**CD138, n (%)**	
**MGUS**	11 (16.18)	**Positive**	61 (89.71)
**SMM**	20 (29.41)	**Negative**	3 (4.41)
**NDMM**	19 (27.94)	**N/A**	4 (5.88)
**RRMM**	18 (26.47)	**cyKAPPA, n (%)**	
**Mspike Value (SPEP), gm/dL**		**Positive**	38 (55.88)
**Median (min-max)**	0.95 (0-8.9)	**Negative**	25 (36.76)
**FLOW Aberrant PCs from total analyzed, %**		**N/A**	4 (5.88)
**Median (min-max)**	95.1 (0-100)	**Indeterminate**	1 (1.47)
**Alive, n (%)**		**cyLAMBDA, n (%)**	
**Yes**	60 (88.24)	**Positive**	14 (20.59)
**No**	8 (11.76)	**Negative**	19 (27.94)
**Free Lambda Light Chain, mg/L**		**N/A**	4 (5.88)
**Median (min-max)**	10.93 (1.65-6060.58)	**Indeterminate**	1 (1.47)
**Free Kappa Light Chain, mg/L**		**Karotype, n (%)**	
**Median (min-max)**	30.335 (0.92-8159.04)	**Abnormal**	12 (17.65)
**Serum Free Light Chain Ratio, i:U**		**Hyperdiploidy**	2 (2.94)
**Median (min-max)**	15.47 (0-1362.11)	**Normal**	40 (58.82)
**IgA, mg/dL**		**N/A**	14 (20.59)
**Median (min-max)**	75 (2-6640)	**t(4;14)/(p16;32), n (%)**	

**IgG, mg/dL**		**Positive**	4 (5.88)
**Median (min-max)**	1101 (10-11361)	**Negative**	58 (85.29)
**IgM, mg/dL**		**N/A**	6 (8.82)
**Median (min-max)**	29.5 (10-475)	**t(14:16)/(q32;q23), n (%)**	
**Bence Jones, mg/total volume in 24 hours**		**Negative**	63 (92.65)
**Median (min-max)**	0 (0-11852)	**N/A**	5 (7.35)
**Serum Immunofixation, n (%)**		**t(11;14)/(q13;q32) IGH/CCND1, n (%)**	
**Positive**	62 (91.18)	**Positive**	13 (19.12)
**Negative**	6 (8.82)	**Negative**	50 (73.53)
**Serum Immunofixation: Heavy chain, n (%)**		**N/A**	5 (7.35)
**IgA**	9 (13.24)	**Deletion 17p/TP53, n (%)**	
**IgD**	1 (1.47)	**Positive**	3 (4.41)
**IgG**	40 (58.82)	**Negative**	60 (88.24)
**IgM**	6 (8.82)	**N/A**	5 (7.35)
**Negative**	12 (17.65)	**Gain 17p/TP53 - trisomy 17, n (%)**	
**Serum Immunofixation: light chain, n (%)**		**Positive**	6 (8.82)
**Kappa**	46 (67.65)	**Negative**	57 (83.82)
**Lambda**	19 (27.94)	**N/A**	5 (7.35)
**Negative**	3 (4.41)	**Gain FGFR3 /trisomy 4, n (%)**	
**Immunoparesis MGUS/SMM only, n (%)**		**Positive**	2 (2.94)
**Yes**	12 (17.65)	**Negative**	61 (89.71)
**No**	19 (27.94)	**N/A**	5 (7.35)
**N/A**	37 (54.41)	**Monosomy 13/ loss RB1/ Del 13, n (%)**	
**Urine Immunofixation: Heavy chain, n (%)**		**Positive**	24 (35.29)
**Negative**	56 (82.35)	**Negative**	5 (7.35)
**Not done**	12 (17.65)	**N/A**	39 (57.35)
**Urine Immunofixation: light chain, n (%)**		**gain CCND1/MYEVO/IGH -trisomy 11, n (%)**	
**Kappa**	25 (36.76)	**Positive**	18 (26.47)
**Lambda**	10 (14.71)	**Negative**	45 (66.18)
**Negative**	21 (30.88)	**N/A**	5 (7.35)
**Not done**	12 (17.65)	**Gain CKS1B (1q21)-trisomy 1, n (%)**	
**Type of BM, n (%)**		**Positive**	16 (23.53)
**Bilateral**	27 (39.71)	**Negative**	47 (69.12)
**Unilateral (Left Side)**	25 (36.76)	**N/A**	5 (7.35)
**Unilateral (Right Side)**	16 (23.53)	**Del of CDKN2C (1q32), n (%)**	
**CD19, n (%)**		**Positive**	4 (5.88)
**Positive**	6 (8.82)	**Negative**	59 (86.76)
**Negative**	58 (85.29)	**N/A**	5 (7.35)
**N/A**	4 (5.88)	**Gain IGH -trisomy 14, n (%)**	
**CD27, n (%)**		**Positive**	5 (7.35)
**Positive**	30 (44.12)	**Negative**	58 (85.29)
**Negative**	34 (50)	**N/A**	5 (7.35)
**N/A**	4 (5.88)	**Cyclin D1 stain, n (%)**	
		**Positive**	8 (11.76)
		**Negative**	39 (57.35)
		**N/A**	21 (30.88)

Supplemental Figure 1. Circulating rare cells detected in the PB of patients diagnosed with MGUS, SMM, NDMM, or RRMM. Boxplot of cellular distribution per disease state. Black line is median, boxed area is the interquartile range (IQR), error-bars show the complete range of the data. D: DAPI, 138: CD138, 45: CD45, Peri: perinuclear localization of BCMA, Memb: membrane bound BCMA.

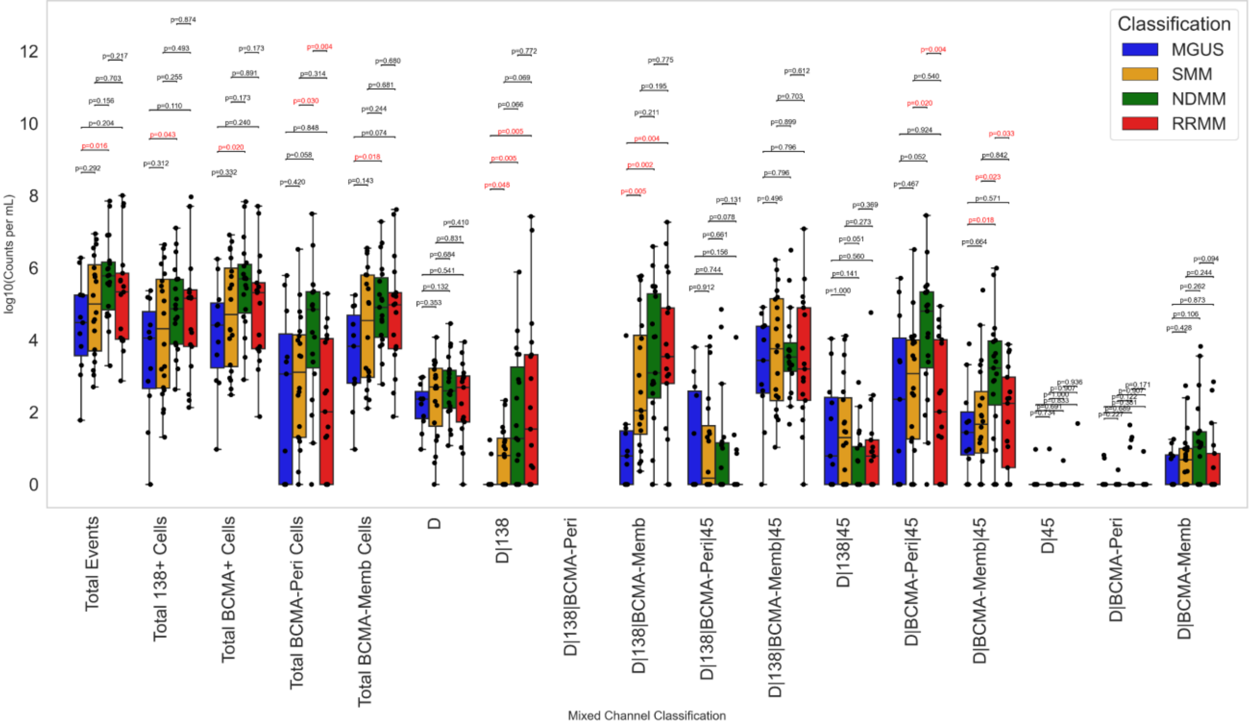

Supplemental Table 2. Univariate logistic regression and multivariate analysis for predicting disease state. Prediction accuracy presented for each disease state comparison by analyte type. D: DAPI, 138: CD138, 45: CD45, Peri: perinuclear localization of BCMA, Memb: membrane bound BCMA.

**Analyte**	MGUS vs. SMM	MGUS vs. NDMM	MGUS vs. RRMM	SMM vs. NDMM	SMM vs. RRMM	NDMM vs. RRMM	Precursor vs. Cancer	Precursor vs. NDMM
Total Events	71.43	83.33	33.33	37.50	62.50	50.00	64.29	50.00
Total 138+ Cells	71.43	66.67	33.33	37.50	75.00	50.00	64.29	60.00
Total BCMA+ Cells	71.43	66.67	33.33	37.50	62.50	12.50	71.43	60.00
Total BCMA-Memb Cells	57.14	83.33	33.33	37.50	75.00	37.50	71.43	70.00
Total BCMA-Peri Cells	71.43	50.00	83.33	62.50	50.00	75.00	50.00	60.00
D	71.43	50.00	66.67	25.00	37.50	75.00	50.00	70.00
D|138	71.43	83.33	50.00	37.50	75.00	62.50	78.57	80.00
D|138|BCMA-Memb	71.43	100.00	50.00	25.00	75.00	50.00	71.43	60.00
D|138|BCMA-Peri|45	71.43	50.00	66.67	37.50	37.50	50.00	42.86	70.00
D|138|BCMA-Memb|45	71.43	33.33	0.00	37.50	50.00	50.00	50.00	60.00

D|138|45	71.43	66.67	66.67	75.00	37.50	37.50	50.00	60.00
D|BCMA-Peri|45	71.43	50.00	83.33	62.50	50.00	75.00	71.43	60.00
D|BCMA-Memb|45	71.43	66.67	66.67	50.00	62.50	75.00	71.43	70.00
D|45	71.43	50.00	66.67	37.50	62.50	62.50	50.00	60.00
D|BCMA-Peri	71.43	50.00	50.00	50.00	62.50	75.00	50.00	70.00
D|BCMA-Memb	71.43	50.00	66.67	37.50	62.50	75.00	42.86	60.00
Decision Tree	71.43	100.00	50.00	87.50	62.50	75.00	85.71	70.00
Gradient Boosting	57.14	83.33	50.00	75.00	50.00	87.50	78.57	70.00
Logistic Regression	57.14	66.67	66.67	50.00	37.50	75.00	57.14	70.00
Neural Network	71.43	50.00	66.67	62.50	62.50	75.00	78.57	60.00
Random Forest	42.86	100.00	33.33	62.50	37.50	75.00	85.71	70.00
SVM	71.43	50.00	66.67	50.00	62.50	75.00	50.00	60.00
